# Dissecting and engineering of the TetR family regulator SACE_7301 for enhanced erythromycin production in *Saccharopolyspora erythraea*

**DOI:** 10.1186/s12934-014-0158-4

**Published:** 2014-11-13

**Authors:** Hang Wu, Meng Chen, Yongrong Mao, Weiwei Li, Jingtao Liu, Xunduan Huang, Ying Zhou, Bang-Ce Ye, Lixin Zhang, David T Weaver, Buchang Zhang

**Affiliations:** Institute of Health Sciences, School of Life Sciences, Anhui University, Hefei, 230601 China; CAS Key Laboratory of Pathogenic Microbiology & Immunology, Institute of Microbiology, Chinese Academy of Sciences, Beijing, 100101 China; State Key Laboratory of Bioreactor Engineering, East China University of Science & Technology, Shanghai, 200237 China; Beijing Institute of Cell Biotechnology, Beijing, 100043 China

**Keywords:** *Saccharopolyspora erythraea*, Erythromycin, SACE_7301, TetR family regulator, Gene overexpression, Metabolic engineering

## Abstract

**Background:**

*Saccharopolyspora erythraea* was extensively utilized for the industrial-scale production of erythromycin A (Er-A), a macrolide antibiotic commonly used in human medicine. Yet, *S. erythraea* lacks regulatory genes in the erythromycin biosynthetic gene (*ery*) cluster, hampering efforts to enhance Er-A production via the engineering of regulatory genes.

**Results:**

By the chromosome gene inactivation technique based on homologous recombination with linearized DNA fragments, we have inactivated a number of candidate TetR family transcriptional regulators (TFRs) and identified one TFR (SACE_7301) positively controlling erythromycin biosynthesis in *S. erythraea* A226. qRT-PCR and EMSA analyses demonstrated that SACE_7301 activated the transcription of erythromycin biosynthetic gene *eryAI* and the resistance gene *ermE* by interacting with their promoter regions with low affinities, similar to BldD (SACE_2077) previously identified to regulate erythromycin biosynthesis and morphological differentiation. Therefore, we designed a strategy for overexpressing *SACE_7301* with 1 to 3 extra copies under the control of PermE* in A226. Following up-regulated transcriptional expression of *SACE_7301*, *eryAI* and *ermE*, the *SACE_7301*-overexpressed strains all increased Er-A production over A226 proportional to the number of copies. Likewise, when *SACE_7301* was overexpressed in an industrial *S. erythraea* WB strain, Er-A yields of the mutants WB/7301, WB/2×7301 and WB/3×7301 were respectively increased by 17%, 29% and 42% relative to that of WB. In a 5 L fermentor, Er-A accumulation increased to 4,230 mg/L with the highest-yield strain WB/3×7301, an approximately 27% production improvement over WB (3,322 mg/L).

**Conclusions:**

We have identified and characterized a TFR, SACE_7301, in *S. erythraea* that positively regulated erythromycin biosynthesis, and overexpression of *SACE_7301* in wild-type and industrial *S. erythraea* strains enhanced Er-A yields. This study markedly improves our understanding of the unusual regulatory mechanism of erythromycin biosynthesis, and provides a novel strategy towards Er-A overproduction by engineering transcriptional regulators of *S. erythraea.*

**Electronic supplementary material:**

The online version of this article (doi:10.1186/s12934-014-0158-4) contains supplementary material, which is available to authorized users.

## Background

*Saccharopolyspora erythraea*, a Gram-positive industrial actinomycetes, produces a valuable macrolide antibiotic erythromycin A (Er-A). Er-A and its semi-synthetic derivatives, such as clarithromycin, azithromycin, dirithromycin, roxithromycin, and telithromycin, are widely used in medicine to treat infections caused by pathogenic Gram-positive bacteria [1]. Considering its high industrial and medical importance, enhancement of Er-A production has been performed by metabolic engineering or classical mutagenesis methods over the past 60 years [2-4]. Also, as a model actinomycete system, *S. erythraea* has been used for studying the biosynthesis and combinatorial biosynthesis of polyketide antibiotics, which are synthesized by modular type I polyketide synthase (PKS) [5,6]. The erythromycin biosynthetic pathway has been investigated by genetic and biochemical approaches [7,8]. The erythromycin biosynthetic gene (*ery*) cluster contains 20 genes arranged in four major polycistronic units, spanning over 60 kb of DNA [9]. Unusually, the *ery* cluster lacks any regulatory genes in *S. erythraea*, hampering efforts to improve Er-A production by modulating gene regulation pathways.

Based on a DNA microarray strategy, the industrial overproducer strain was found to express the entire *ery* cluster several days longer than the wild-type strain, suggesting that there exist some regulators for controlling erythromycin biosynthesis [10]. *In vitro* and *in vivo* approaches led to the identification of BldD (SACE_2077), a key developmental regulator in actinomycetes [11], controlling erythromycin biosynthesis and morphological differentiation in *S. erythraea* [12]. BldD was found to bind to the promoter regions of *ery* cluster. However, the action of BldD on the promoters of *ery* genes was much weaker than on its own promoter [12]. Recently, a putative regulatory protein, SACE_5599, was proved to positively control erythromycin production and morphological differentiation in *S. erythraea* [13]. Nevertheless, the knowledge of molecular regulatory mechanisms controlling erythromycin biosynthesis remains limited.

The TetR family transcriptional regulators (TFRs), containing an N-terminal HTH DNA-binding motif and a C-terminal ligand recognition domain, are widely distributed in bacteria, playing important roles in antibiotic biosynthesis, efflux pumps, osmotic stress, and other functions [14]. TFRs regulate the biosynthesis of multiple antibiotics or morphogenesis of actinomycetes [15-28], suggesting that certain TRFs from *S. erythraea* are candidates for a role in erythromycin biosynthesis. With the availability of the complete genome sequence, 101 putative TFRs in *S. erythraea* were inferred [29]. Based on the homologous recombination with linearized DNA fragments to inactivate specific genes, a number of the *S. erythraea* TFRs were examined, resulting in the identification of two TFRs (SACE_7040 and SACE_0012) having an association with morphological differentiation [30,31], and one TFR (SACE_3986) negatively controlling the erythromycin biosynthesis [32]. Nevertheless, functions of most TFRs in *S. erythraea* remain to be elucidated.

In this study, we identified and characterized a novel TFR (SACE_7301) that positively regulated erythromycin biosynthesis. SACE_7301 was found to increase the transcription of erythromycin biosynthetic gene *eryAI* and the resistance gene *ermE* by binding to their promoter regions in *S. erythraea.* Further overexpression of *SACE_7301* led to enhanced Er-A titers in the wild-type *S. erythraea* A226 and the high-yield industrial strain WB.

## Results

### SACE_7301 positively regulates the erythromycin biosynthesis

**Figure 1:**
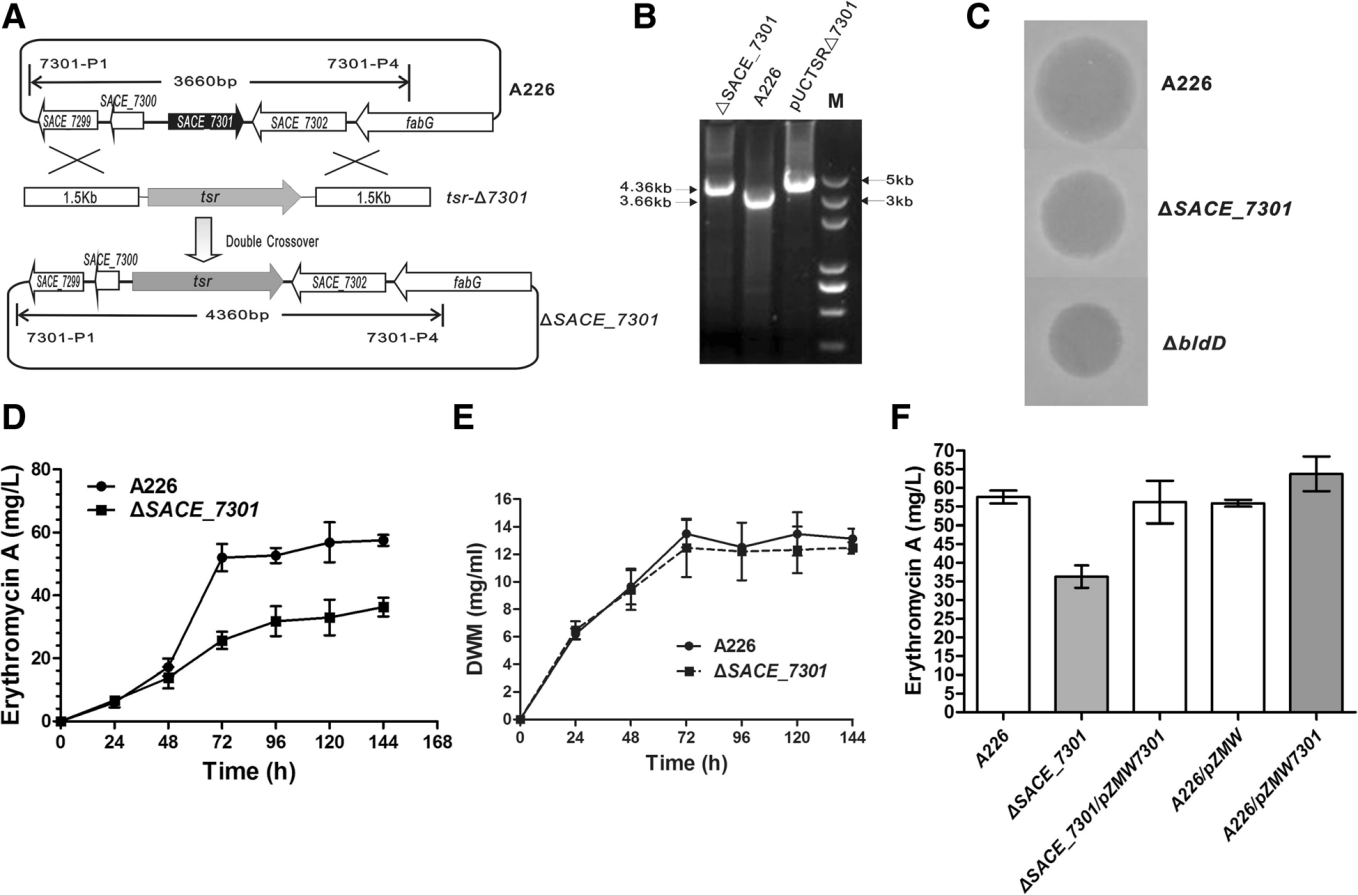
**Inactivation of** ***SACE_7301*** **in** ***S. erythraea*** **A226. (A)** Schematic inactivation of *SACE_7301* in A226 by linearized fragment homologous recombination. **(B)** Confirmation of the Δ*SACE_7301* mutant by PCR analysis using the primers 7301-P1 and 7301-P4. Lanes: M, 5000-bp DNA ladder. The size of 3,660 bp for a PCR-amplified band was detected in A226, while the bands of the size 4,360 bp were observed in pUCTSR-Δ7301 and Δ*SACE_7301*, indicating that *SACE_7301* was replaced with *tsr*. **(C)** Inhibition tests of A226, Δ*SACE_7301* and Δ*bldD* fermentation broths against *B. subtilis* PUB110. **(D)** Time course of Er-A yield in A226 and Δ*SACE_7301* by HPLC analysis; Mean values of at least three replicates were shown, with the standard deviation indicated by error bars. **(E)** Growth curves of A226 and Δ*SACE_7301*. The two strains were cultured in the R5 liquid fermentation medium, and their dry weights of mycelia (DWM) were measured. **(F)** Er-A production in A226, Δ*SACE_7301*, Δ*SACE_7301*/pZMW7301, A226/pZMW, and A226/pZMW7301 cultured in R5 liquid fermentation medium for 6 days by HPLC analysis. Mean values of at least three replicates were shown, with the standard deviation indicated by error bars.

Since extensive investigations have proved that TFRs are involved in the antibiotic biosynthesis in actinomycetes through gene inactivation and bioassay experiments [33], we have identified several TFRs pertinent to erythromycin production in *S. erythraea*, including the repressor SACE_3986 [32], and the activator SACE_7301 currently studied. By homologous recombination of linearized fragments (Figure 1A), *SACE_7301* was replaced by the thiostrepton resistance gene (*tsr*) in *S. erythraea* A226, and the desired mutant, named as Δ*SACE_7301,* was isolated and confirmed by PCR analysis (Figure 1B).

Erythromycin titers of A226 and Δ*SACE_7301* were estimated by fermentation and bioassay. Δ*SACE_7301* displayed lower inhibitory activity against *Bacillus subtilis* relative to A226, but had slightly higher levels than the *bldD* deletion mutant (Figure 1C), a previously identified regulator of erythromycin biosynthesis [12]. Furthermore, A226 and Δ*SACE_7301* were cultivated at 30°C for up to 6 days in the R5 liquid fermentation medium, and the extracts of those cultures were analyzed by HPLC. Compared with the parental strain A226, Δ*SACE_7301* had a 38% reduction in the Er-A yield in 6 day fermentation period (from 58 mg/L to 36 mg/L). (Figure 1D). However, the two strains had comparable growth rates and cell densities (Figure 1E). Thus, SACE_7301 is postulated to positively regulate erythromycin production in *S. erythraea*.

In order to exclude the possibility that the decreased erythromycin production was due to a random mutation in other chromosome loci of *S. erythraea*, Δ*SACE_7301* was complemented with a copy of *SACE_7301* (pZMW7301). The Er-A yield of the complemented strain Δ*SACE_7301*/pZMW7301 was nearly recovered to the parental level (Figure 1F). To further substantiate the role of SACE_7301 as an activator for erythromycin production, we introduced pZMW and pZMW7301 into A226, obtaining the control strain A226/pZMW and the *SACE_7301*-overexpressed strain A226/pZMW7301, respectively. As detected by HPLC analysis, the Er-A yield of A226/pZMW-7301 was 64 mg/L, that was 10% and 14% increased relative to those of A226 (58 mg/L) and A226/pZMW (56 mg/L) respectively (Figure 1F)*.* Taken together*,* our results verified that SACE_7301 acted as a positive regulator controlling the erythromycin production in *S. erythraea*.

### SACE_7301 is not involved in the morphological differentiation of *S. erythraea*

Previously, BldD and SACE_5599 were identified to concurrently regulate erythromycin biosynthesis and morphological differentiation in *S. erythraea* [12,13]. In order to discern whether SACE_7301 was also associated with the morphological differentiation, we inoculated A226 and its derivative mutants on R3M agar medium for observing sporulation. Δ*SACE_7301*, Δ*SACE_7301*/pZMW7301 and A226/pZMW7301 on R3M plates were identical to A226 in the rate of aerial mycelia formation, whereas Δ*bldD* showed the defect of aerial hyphae as expected (data not shown). These results further substantiate that SACE_7301 acts exclusively as a positive regulator for the synthesis of erythromycin in *S. erythraea*.

### Overexpression of *SACE_7301* increases the transcription of *eryAI* and *ermE*

**Figure 2:**
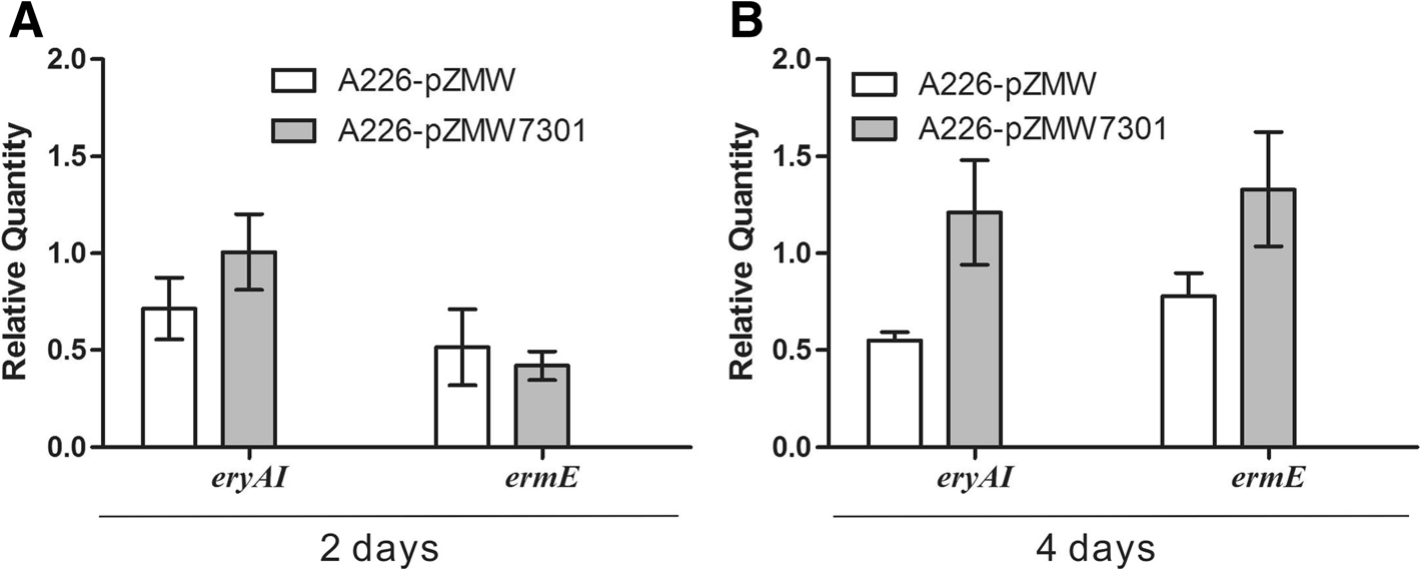
**Effects of** ***SACE_7301*** **overexpression on transcriptional levels of** ***eryAI*** **and** ***ermE*** **.** qRT-PCR was used to quantify the amounts of transcripts produced by A226/pZMW, and A226/pZMW7301 cultured in R5 fermentation medium for 2 **(A)** or 4 days **(B)**. Mean values of at least three replicates were shown, with the standard deviation indicated by error bars.

To investigate the effect of *SACE_7301* overexpression on the transcription of *ery* genes, we chose erythromycin biosynthetic gene *eryAI* (encoding polyketide synthase I) and the resistance gene *ermE* (encoding rRNA methyltransferase) for transcriptional comparison between control strain A226/pZMW and overexpressed strain A226/pZMW7301 on the second or fourth day of growth. When grown for 4 days, A226/pZMW7301 exhibited 2.2- and 1.7- folds transcription increases detected by qRT-PCR for *eryAI* and *ermE* in comparison to A226/pZMW (Figure 2B). Similar transcriptional levels of *eryAI* and *ermE* were found for the two strains in 2-day growth (Figure 2A). The data supports the interpretation that SACE_7301 plays a positive role in the erythromycin biosynthesis by increasing the transcription of several structural and resistance genes.

### SACE_7301 binds to the promoter regions of *eryAI* and *ermE*

TetR family members often regulate the transcription of target genes by specifically binding to their promoters [33]. To examine whether SACE_7301 might directly regulate the transcription of *eryAI* and *ermE*, a full-length SACE_7301 gene was expressed in *Escherichia coli* BL21 (DE3) using pET22b, and the purified SACE_7301 protein fused with His tag (Additional file 1: Figure S1) was used for *in vitro* binding experiments by EMSA.

**Figure 3:**
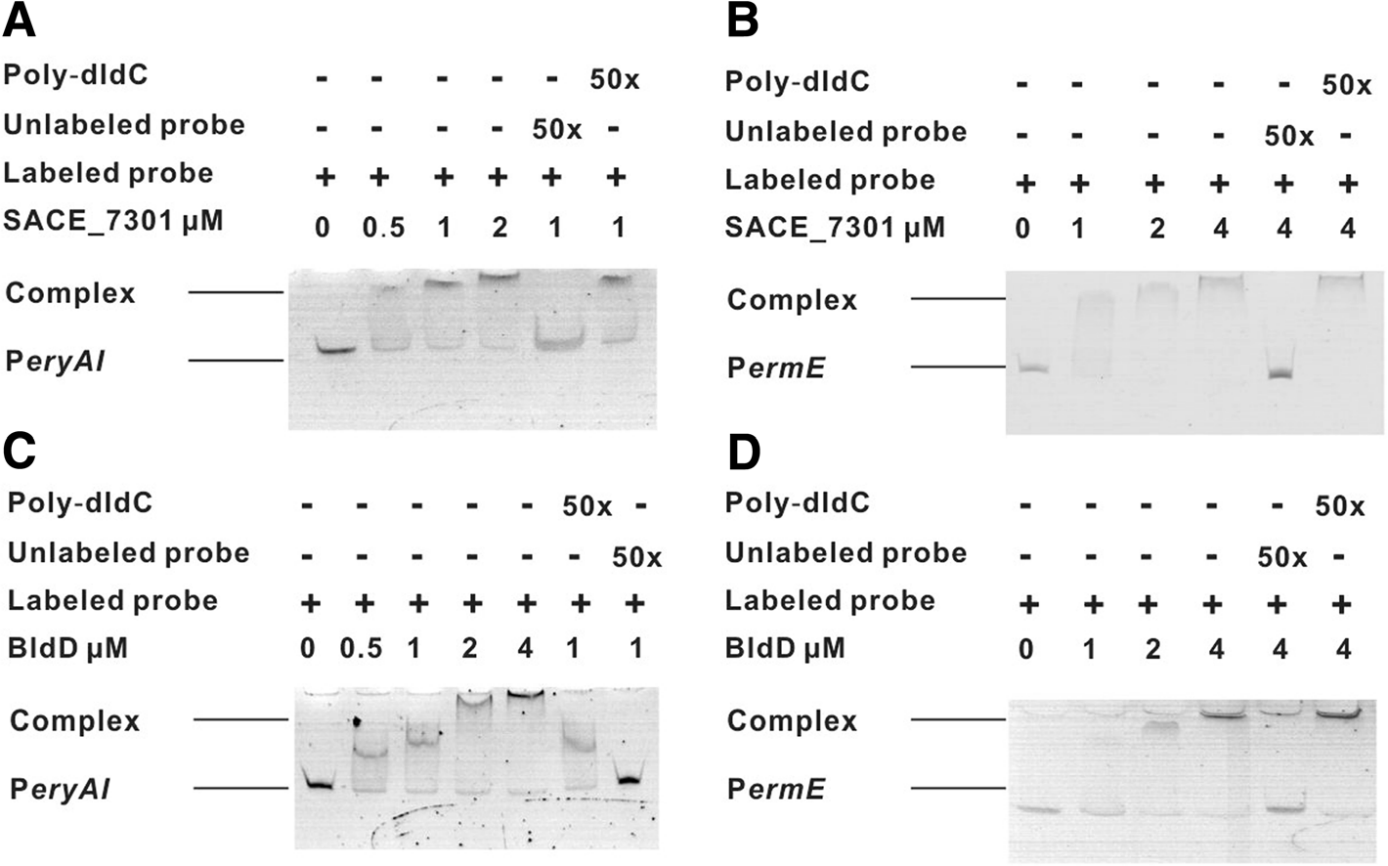
**Electrophoretic mobility shift assays (EMSAs) with the promoter regions of** ***eryAI*** **or** ***ermE*** **and purified His**_**6**_ **-tagged SACE_7301 or His**_**6**_ **-tagged BldD. (A)** EMSAs with recombinant SACE_7301 protein and P*eryAI*. **(B)** EMSAs with recombinant SACE_7301 protein and P*ermE*. **(C)** EMSAs with recombinant BldD protein and P*eryAI*. **(D)** EMSAs with recombinant BldD protein and P*ermE*. Each of the lanes contained 10 ng DNA. Results were one representative example of at least three replicates.

6-FAM labeled *eryAI* and *ermE* promoter DNA fragments were respectively mixed with purified His_6_-tagged SACE_7301. At concentrations equal or greater than 1 μM of SACE_7301, mobility shifts were evident. Meantime, it was found that a 50-fold excess of unlabeled probes markedly competed with labeled probes for binding to SACE_7301 (Figure 3A-B). As a negative control, a non-specific DNA, poly dIdC, was used to compete with the 6-FAM labeled probes, and the shifted bands did not disappear (Figure 3A-B), confirming that SACE _7301 specifically bound to the promoter regions of *eryAI* and *ermE*.

To evaluate the relative affinity of SACE_7301 to above-mentioned probes, we expressed recombinant BldD protein in *E. coli* BL21 (DE3), and purified it with His_6_-tag (Additional file 1: Figure S1). EMSAs showed that the concentrations of BldD shifting the two promoter probes of *eryAI* and *ermE* were similar to that of SACE_7301 (Figure 3C-D), indicating that SACE_7301 and BldD had the same degree of affinities to the two promoters. Therefore, the combined data indicates that SACE_7301 up-regulates the transcription of *eryAI* and *ermE* by directly interacting with their promoter regions.

### Effects of *SACE_7301* overexpression on erythromycin biosynthesis in *S. erythraea* A226

**Figure 4:**
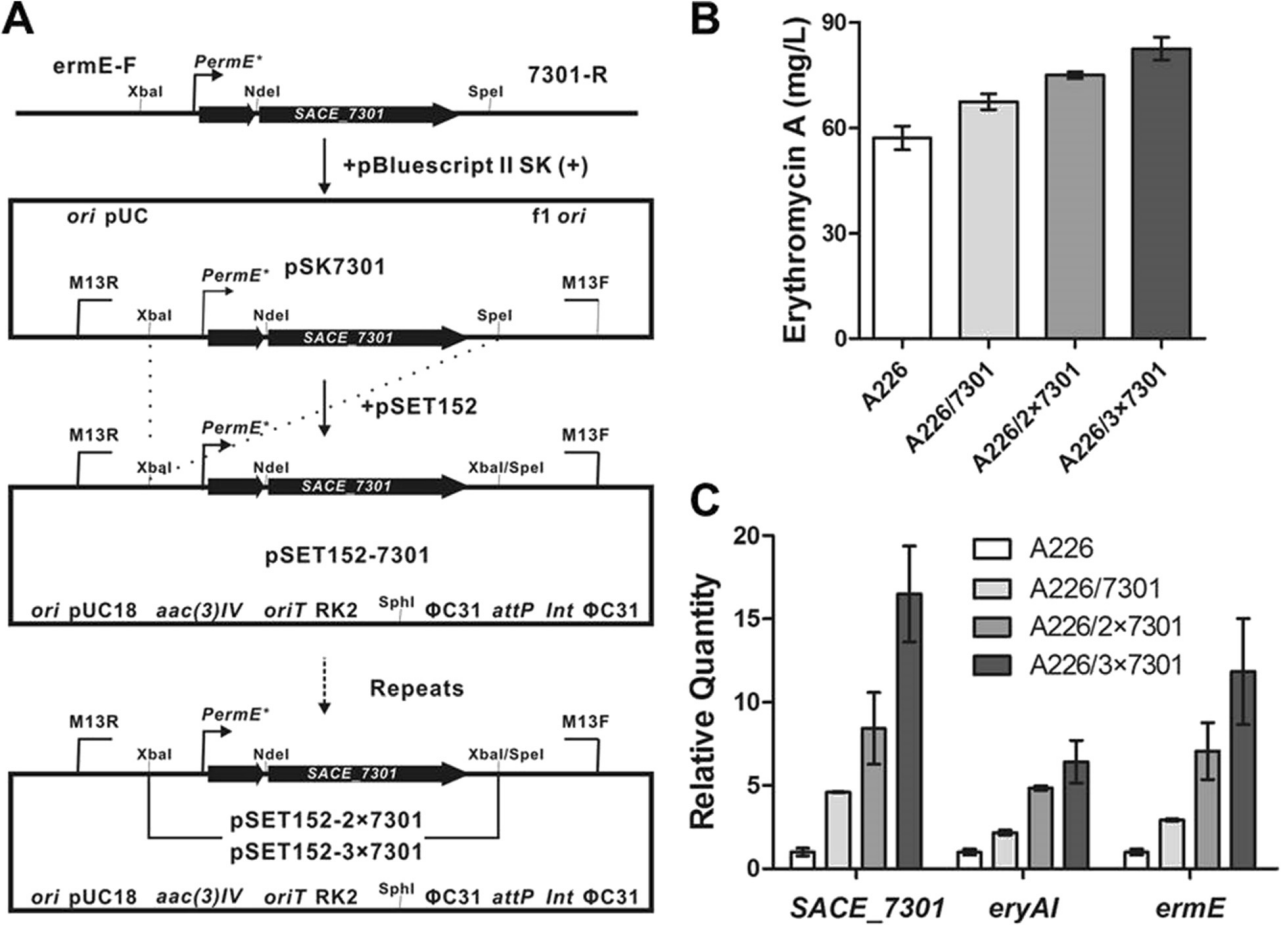
**Effects of overexpression of** ***SACE_7301*** **on erythromycin biosynthesis in** ***S. erythraea*** **A226. (A)** Schematic illustration of the strategy for construction of expression plasmids with 1 to 3 copies of *SACE_7301*. **(B)** Er-A production of A226 and *SACE_7301*-overexpressed mutants. **(C)** qRT-PCR analyses of gene transcription in A226 and *SACE_7301*-overexpressed mutants. Mean values of at least three replicates were shown, with the standard deviation indicated by error bars.

As overexpression of *SACE_7301* with an extra copy displayed an increase of Er-A production in *S. erythraea* A226 as described above, we proposed that increased copy numbers of *SACE_7301* would lead to additional increases in Er-A levels. *SACE_7301* under the control of PermE* was cloned into pSET152, creating 1, 2, or 3 copies of *SACE_7301*, introduced into A226 (Figure 4A), to generate A226/7301, A226/2×7301 and A226/3×7301 strains respectively. By fermentation and HPLC analysis, the three strains all presented Er-A product improvement (Figure 4B). Furthermore, the Er-A production exhibited a positive correlation with the copy number of *SACE_7301*, reaching the highest 1.44 folds with 3 copies (Figure 4B). When cultured in the R5 liquid medium, these strains had comparable growth rates and cell densities (Additional file 1: Figure S2), suggesting that the enhanced erythromycin yield in *SACE_7301*-overexpressed strains was not caused by the increase of biomass.

In addition, qRT-PCR was used to compare the transcriptional levels of *SACE_7301*, *eryAI* and *ermE* between A226 and its *SACE_7301*-overexpressed strains. With the increase of *SACE_7301* copy number, the transcripts of *SACE_7301* in A226/7301, A226/2×7301 and A226/3×7301 were found to be up-regulated by 4.6-, 8.4- and 16.5-folds relative to that of A226 (Figure 4C). Likewise, the transcripts of *eryAI* and *ermE* in those mutants were gradually increased compared to the original level (Figure 4C). These results indicated that overexpression of *SACE_7301* with 1 to 3 extra copies in *S. erythraea* associated with a stepwise increase of Er-A production by promoting the transcription of *eryAI* and *ermE*. In conjunction with RNA changes, A226/3×7301 also demonstrated increased resistance to erythromycin over A226 when cultured for 2 days with addition of greater than 160 μg/ml erythromycin (Additional file 1: Figure S3).

Also, we cultured A226/3×7301 in the R5 liquid medium for 6 days without addition of apramycin, and found that the size of the gene expression cassette containing the 3 copies of *SACE_7301* from plasmid pSET152-3×7301 did not change by PCR analysis (Additional file 1: Figure S4), indicating that the engineered strain had a stably inserted *SACE_7301*.

### Improved Er-A production by overexpression of *SACE_7301* in an industrial strain WB

**Figure 5:**
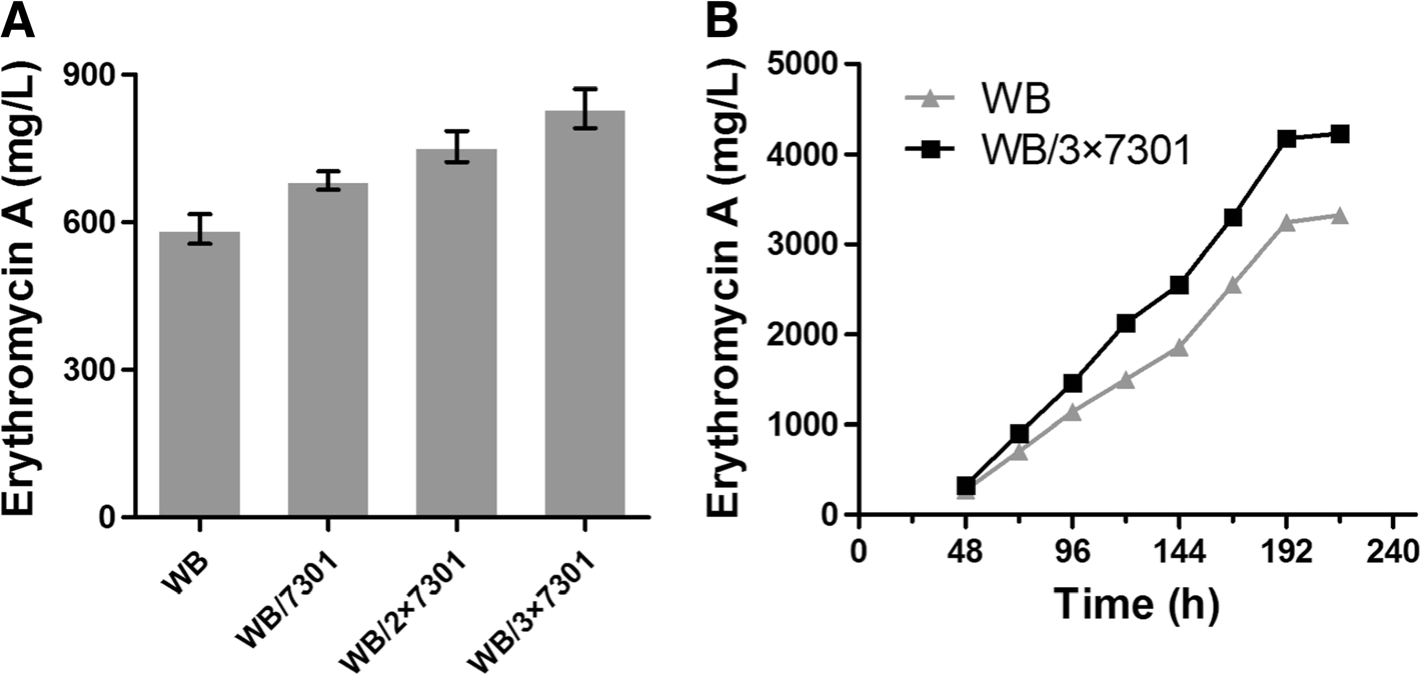
**Overexpression of** ***SACE_7301*** **in the industrial strain** ***S. erythraea*** **WB resulted in a higher Er-A production. (A)** Er-A yield of WB and its derivatives cultured in flasks for 6 days. Mean values of at least three replicates were shown, with the standard deviation indicated by error bars. **(B)** Time course of Er-A production of WB and WB/3×7301 in a 5 L fermentor. One of the representative datasets was shown.

To examine the applicability of overexpression of *SACE_7301* for enhancing erythromycin production in industrially-relevant strains, *SACE_7301* was introduced into an industrial *S. erythraea* WB strain with 1, 2 or 3 extra copies under the control of PermE*, and the desired mutants WB/7301, WB/2×7301 and WB/3×7301 were obtained. In flask experiments with an industrial medium for 6 days, the Er-A yields of WB/7301 (685 mg/L), WB/2×7301 (755 mg/L) and WB/3×7301 (832 mg/L) were respectively increased by 17%, 29%, and 42% in comparison to that of WB (586 mg/L) (Figure 5A). Likewise, no significant change of cell growth was detected for these strains in comparison (Additional file 1: Figure S2).

We also tested the reproducibility of one of the engineered strains in a larger scale by selecting the highest-yield strain WB/3×7301. Following 8-day fermentation at a 5 L fermentor scale, WB/3×7301 exhibited a strikingly higher Er-A yield (4,230 mg/L), that was 27% greater than WB (3,322 mg/L). Since this strain maintains the characteristics of its parental strain, we conclude that the additional Er-A production from *SACE_*7301-engineered industrial strains will be of significant commercial value.

## Discussion

TFRs form homodimers that generally act as transcriptional repressors to regulate the transcription of genes involved in antibiotic biosynthesis or resistance [34], but little is known about the TFR as a positive regulator for controlling the antibiotic biosynthesis in actinomycetes. Recently, the TetR family regulatory gene *gouR*, situated in the gene cluster of gougerotin biosynthesis, was characterized to be a positive regulator for modulating the gougerotin production by coordinating its biosynthesis and export in *Streptomyces graminearus* [27]. In this work, we identified a novel TFR (SACE_7301) from *S. erythraea*, and proved that it played a positive role in regulating erythromycin biosynthesis.

SACE_7301 was shown to specifically bind to the promoter regions of *eryAI and ermE in vitro*, and enhance their transcription. *SACE_7301*, located approximately 8.1 Mb in the chromosome of *S. erythraea*, is not closely positioned with the *ery* cluster (GenBank Accession No. NC-009142. 778,214–832,825 nt, 0.8 Mb) and therefore it became evident that the gene product of SACE_7301 might function as a trans-acting factor remotely regulating erythromycin biosynthesis. This regulation pattern may be similar to that of BldD, a previously identified regulator for erythromycin biosynthesis [12]. As SACE_7301 exhibited low affinities to *eryA* and *ermE* promoters, it was difficult to determine the tight binding sites through DNase I footprinting assay. On the other hand, moderate increase in Er-A production was achieved by overexpression of *SACE_7301* with an extra copy, similar to BldD in *S. erythraea* A226 [35]. These data indicate that SACE_7301 is likely a positive regulator that directly acts on the promoters of *ery* genes.

Through phylogenetic analysis [34], SACE_7301 homologs are distributed in rare actinomycetes, such as KUTG_05296 from *Kutzneria* sp. 744 (62% identity), Amir_6979 from *Actinosynnema mirum* ATCC29888 (63% identity), BN6_83740 from *Saccharothrix espanaensis* ATCC51144 (60% identity) (Additional file 1: Figure S5). However, since none of these TFRs has been uncovered in actinomycetes, it potentially signifies a novel regulatory mechanism in actinomycetes, such as how it functions with its ligand [36].

To further improve the production of erythromycin, an overexpression tactic was designed and utilized for overexpressing *SACE_7301* under PermE* in wild-type strain A226 and industrial overproducer WB. Along with the increase of *SACE_7301* copy number, *SACE_7301*, *eryAI* and *ermE* were significantly up-regulated, and Er-A yields of those mutants rose step by step. However, these changes in transcription level do not linearly correspond to the changes in Er-A production. A similar result was also reported for the TetR family regulator GouR in *S. graminearus* [27].

Overexpression of *bldD* or *SACE_5599* could improve erythromycin production in the wild-type *S. erythraea* strain, but not in the industrial overproducer [13]. Considering that WB is already an industrial strain, it is likely that overexpression of *SACE_7301* will have commercial value when applied in other erythromycin high-yield *S. erythraea* strains. In other examples, the overexpression approach was utilized to engineer the halogenase gene *ctcP*, resulting in improvement of chlortetracycline production in industrial *S. aureofaciens* [37]. Overexpression of tailor genes *eryK* and *eryG* was also performed to enhance the production and purity of Er-A in an industrial *S. erythraea* [4]. To our knowledge, this is the first report for tandemly expressing transcriptional regulators to enhance antibiotic production in actinomycetes. We anticipate that the strategy of overexpression of additional regulators will be generally applicable for improvement of other antibiotics in industry.

## Conclusion

The novel TFR SACE_7301 positively regulates erythromycin biosynthesis in *S. erythraea*, and it activated the transcription of erythromycin biosynthetic gene *eryAI* and the resistance gene *ermE* by interacting with their promoters. Furthermore, when *SACE_7301* was overexpressed in wild-type or industrial *S. erythraea* strains with 1 to 3 extra copies, Er-A yields had stepwise enhancement in comparison to the parental strains. Our data here form a new understanding of the unusual regulatory mechanism of erythromycin biosynthesis, and provide a valuable means to improve erythromycin production.

## Materials and methods

### Strains, plasmids, and culture conditions

All strains and plasmids used in this study are listed in Table 1. *S. erythraea* and its derivatives were grown either in TSB liquid medium for seed culture, DNA extraction and protoplast preparation, or on R3M agar medium for protoplast regeneration or phenotypic observation with thiostrepton or apramycin when appropriate [38,39]. *E. coli* and *B. subtilis* strains were cultured in Luria-Bertani (LB) liquid medium or on LB plates at 37°C [40]. The general techniques in *E. coli* and *S. erythraea* were performed as described [38,40].

**Table 1:** **Strains and plasmids used in this study**

**Strains or plasmids**	**Description**	**Sources**
***S. erythraea***		
A226	CGMCC 8279, an erythromycin low producer	China Pharmaceutical Culture Collection
Δ*SACE_7301*	A226 with *SACE_7301* deleted	This study
Δ*SACE_7301/*pZMW	Δ*SACE_7301* carrying pZMW	This study
Δ*SACE_7301/*pZMW7301	Δ*SACE_7301* carrying pZMW7301	This study
A226*/*pZMW	A226 carrying pZMW	This study
A226*/*pZMW7301	A226 carrying pZMW7301	This study
A226/7301	A226 carrying pSET152-7301	This study
A226/2×7301	A226 carrying pSET152-2×7301	This study
A226/3×7301	A226 carrying pSET152-3×7301	This study
WB	CGMCC 8280, an erythromycin industrial overproducer	Anhui Wanbei Pharmaceutical Co., Ltd.
WB/7301	WB carrying pSET152-7301	This study
WB/2×7301	WB carrying pSET152-2×7301	This study
WB/3×7301	WB carrying pSET152-3×7301	This study
***E. coli***		
DH5α	F *recA lacZ*M15	[40]
BL21(DE3)	F-*ompT hsd*SB (*rB*-*mB*-) *gal dcm* (DE3)	Novagen
**Plasmids**		
pUCTSR	pUC18 derivative containing a 1.36-kb fragment of a thiostrepton resistance cassette in the BamHI/SmaI sites	[41]
pUCTSR-Δ7301	pUCTSR carrying two 1.5-kb fragments of the flanking sequence of SACE_7301 gene	This study
pZMW	*E. coli*-*S. erythraea* integrative shuttle vector carrying PermE*	[42]
pZMW7301	pZMW carrying *SACE_7301*	This study
pBluescript II SK (+)	*bla*, *lacZ orif1*	Stratagene
pSK-7301	pBluescript II SK (+) carrying *SACE_7301* under the control of PermE*	This study
pSET152	*E. coli*-*S. erythraea* integrative shuttle vector	[43]
pSET152-7301	pSET152 carrying *SACE_7301* under the control of PermE*	This study
pSET152-2×7301	pSET152 carrying two extra copies of *SACE_7301* under the control of PermE*	This study
pSET152-3×7301	pSET152 carrying three extra copies of *SACE_7301* under the control of PermE*	This study
pET22b	T7 promoter, His-tag, *kan*	Novagen
pET22b-7301	pET22b carrying *SACE_7301*	This study
pET22b-bldD	pET22b carrying *bldD*	This study

### Gene inactivation, complementation and overexpression of SACE_7301

With *S. erythraea* A226 genomic DNA as a template, two 1.5-kb DNA fragments flanking the *SACE_7301* gene were amplified by PCR using the primer pairs 7301-P1/7301-P2 and 7301-P3/7301-P4 (Table 2). The two PCR products were respectively digested with EcoRI/KpnI and XbaI/HindIII, and ligated into the corresponding sites of pUCTSR [30], obtaining pUCTSR-Δ7301. By the homologous recombination with linearized fragments [41]*,* the *SACE_7301* gene of *S. erythraea* A226 was replaced with the thiostrepton resistance gene (*tsr*). The desired thiostrepton-resistant mutant, named Δ*SACE_7301*, was further confirmed by PCR analysis using the primers 7301-P1/7301-P4 (Table 2).

Using the primers 7301-P5 and 7301-P6 (Table 2), a 0.66-kb DNA fragment containing a full-length *SACE_7301* was amplified by PCR with the genomic DNA of A226 as a template. The PCR product was cleaved with NdeI/EcoRV, and inserted into the corresponding sites of integrative plasmid pZMW [42], yielding pZMW-7301. By PEG-mediated protoplast transformation, pZMW7301 was introduced into the Δ*SACE_7301* mutant and the parental strain A226, respectively. The complemented strain Δ*SACE_7301*/pZMW7301 and overexpression strain A226*/*pZMW7301 were obtained by apramycin resistance screening and confirmed by PCR analysis with the primers apr-F and apr-R (Table 2).

### Overexpression of *SACE_7301* in *S. erythraea*

In order to introduce 1 to 3 extra copies of *SACE_7301* into the chromosome of *S. erythraea*, the combined DNA fragment containing PermE* and *SACE_7301* from the plasmid pZMW7301 was amplified using the primer pair ermE-F and 7301-R (Table 2). The amplified product was cloned into pBluescript II SK (+) via XbaI and SpeI sites (Figure 4A), and the correct sequence was confirmed by DNA sequencing. The fragment digested with XbaI and SpeI was repeatedly subcloned into XbaI site of pSET152 to generate pSET152-7301, pSET152-2×7301 and pSET152-3×7301, respectively (Figure 4A). Those plasmids were successively introduced into *S. erythraea* A226 or WB, and corresponding *SACE_7301*-overexpressed strains were obtained by apramycin screening and PCR confirmation.

### Fermentation and erythromycin assay

For flask fermentation of A226 and its derivatives, spores from R3M plates cultured for 4 days were incubated in 250 ml baffled flasks containing 30 ml TSB medium for 2 days at 30°C. Then, 3 ml cultures were inoculated into 250 ml baffled flasks containing 30 ml R5 liquid fermentation medium [38], and shaken for 6 days at 30°C (220 rpm). For flask fermentation of the erythromycin high-yielding strain WB and its derivatives, a liquid industrial medium was used as previously described [32]. After 24 h fermentation, 0.3 ml of n-propanol was added into the cultures and further incubated for 5 days at 30°C (220 rpm).

For bioreactor cultures of WB and its *SACE_7301*-overexpressed strain WB/3×7301, the fermentation experiments were carried out in a 5 L fermentor (Baoxing, Shanghai, China) containing 3 L liquid industrial medium. Seed cultures for the cultivation in bioreactors were prepared as described above, using 450 ml industrial seed medium in 2 L baffled flasks. Bioreactors were inoculated with 30 vol.% seed culture. Dissolved oxygen concentration and pH were monitored using autoclavable electrodes (Mettler Toledo, Switzerland). Dissolved oxygen was maintained about 50% with increasing agitation and aeration rate during the bioprocess, and the pH was kept at 7.0 to 7.2. Foaming was controlled by automatic addition of antifoam. Samples (50 ml) were taken every 24 h for Er-A yield analysis with HPLC as described previously [44]. HPLC analysis was performed on Agilent 1260 HPLC system equipped with an Agilent Extend-C18 column (5 μm, 250 × 4.6 mm), which was equilibrated with 40% solution A (acetonitrile, chromatographic grade) and 60% solution B (potassium dihydrogen phosphate, 0.032 mol/L, pH 6.8). An isocratic program was carried out at a flow rate of 1.0 ml/min at 29°C using UV detector at 210 nm.

For bioassay-based analysis, A226, Δ*SACE_7301* and Δ*bldD* were grown in 30 ml R5 liquid medium in 250 ml flasks for 6 days at 30°C. 5 μl fermentation supernatants from those liquid cultures were added to LB agar plates, which were sprayed with an overnight culture of *B. subtilis* PUB110. The plates were incubated at 37°C for 12 h, and the erythromycin production was estimated by detecting the growth-inhibition zones as previously demonstrated [31]. Moreover, Er-A produced by A226, WB and their derivatives were quantitatively analyzed by HPLC.

### Transcriptional analysis by quantitative real-time PCR (qRT-PCR)

The relative transcriptional levels of *eryAI* and *ermE*, from erythromycin biosynthetic gene cluster [7], were determined by qRT-PCR analysis. Specific primers were designed as listed in Table 2. According to the manufacturer’s instructions of a RNA extraction/purification kit (SBS), total RNA was extracted from *S. erythraea* A226 and its derivatives after 2 or 4 days of growth on R5 agar medium [38]. Isolated RNA was treated with DNase I (MBI Fermentas), and reverse transcription was achieved using a cDNA synthesis kit (MBI Fermentas). Quantitative real time PCR reactions were performed on the Applied Biosystems Step-One Plus system with Maxima™ SYBR Green/ROX qPCR Master Mix (MBI Fermentas). The *hrdB* gene encoding the major sigma factor in *S. erythraea* was used as an internal control, and relative quantification was evaluated using a comparative cycle threshold (C_T_) method [45].

**Table 2:** **Primers used in this study**

**Name**	**Sequence (5**′-**3**′**) (restriction site with italic formatting)**	**Use**
7301-P1	CGC*GAATTC*GCATCCTCG AGCACTTCACCG (EcoRI)	Inactivation of *SACE_7301*
7301-P2	CCC*GGTACC*CGGCTCCGGATGGAACCG (KpnI)	
7301-P3	CTC*TCTAGA*CACCACGGCGGCCTGCCG GC (XbaI)	
7301-P4	CCA*AAGCTT*GGAGTCGGCGCATGCTGGTCTC (HindIII)	
7301-P5	AGA*CATATG*ATGAAGGCCGACGTGGAGCAC (NdeI)	Complementation and overexpression of *SACE_7301*
7301-P6	GCA*GATATC*TCACTCCGGTTT CCAGTCGCG (EcoRV)	
apr-F	GCTCATCGGTCAGCTTCTCA	
apr-R	TCGCATTCTTCGCATCCC	
7301-P7	GCTGGGTGTACTCGAAGAACGA	qRT-PCR analysis of *SACE_7301*
7301-P8	TGGGGTCGAAGGAGGAGC	
7301-P9	AGA*CATATG*ATGAAAGCCGATGTGGAGCATTCTGATCGTCCGCGTCCGCGTACCAAGCGGCTGCCGCGC (NdeI)	Expression of SACE_7301 In *E. coli*
7301-P10	CCC*AAGCTT*CTCCGGTTTCCAGTCGCGGCC (HindIII)	
bldD-F	AAA*CATATG*ATGGGCGACTACGCCAAGGCGCTGGG (NdeI)	Expression of BldD In *E. coli*
bldD-R	TGT*AAGCTT*CTCCTCCCGGGCCGGGCGC (HindIII)	
ermE-F	TAA*TCTAGA*GCGAGTGTCCGTTCGAGTGG (XbaI)	Cloning of combined DNA fragment containing PermE* and *SACE_7301*
7301-R	GCA*ACTAGT*TCACTCCGGTTTCCAGTCGCG (SpeI)	
eryAI-P1	CCGCTGATGCCGAACGAC	qRT-PCR analysis of *eryAI*
eryAI-P2	CACCCTTCCCCGCACTCTG	
eryAI-P3	CGGAGCATTTGCTCGCTTTCCAGG	EMSA of *eryAI* promoter
eryAI-P4	GCGTCCCCCTACTCGACGACCAC	
ermE-P1	CCTCCAGGCACCAGTCCAC	qRT-PCR analysis of *ermE*
ermE-P2	AGTCGTTGCGGGAGAAGCT	
ermE-P3	GCGAGTGTCCGTTCGAGTGGCGG	EMSA of *ermE* promoter
ermE-P4	CGCTGGATCCTACCAACCGGCAC	
hrdB-F	GGTCACGCCGTAGACCTGGC	qRT-PCR analysis of *hrdB* as internal reference
hrdB-R	CGGTGTCGTTCACGCTGCTG	
Test-F	GCCAGTGCCAAGCTTGGGCTGCAGGTCGAC	PCR analysis of the cassette containing 3 copies of PermE*-*SACE_7301*
Test-R	GAATTCGATATCGCGCGCGGCCGCGGATCC	

### Overexpression and purification of SACE_7301 and BldD

As the original *SACE_7301* gene could not be expressed in *E. coli* BL21 (DE3) initially, we optimized the N-terminal codons during the amplification of *SACE_7301* using the primers 7301-P9 and 7301-P10 (Table 2). The PCR product was cloned into NdeI and HindIII restriction sites of pET22b to generate pET22b-7301. Then, pET22b-7301 was introduced into *E. coli* BL21 (DE3) for protein production. Gene expression was induced with 1 mM isopropylthiogalactoside (IPTG) for 5 h at 30°C. Cells were harvested and followed by sonication treatment. Purification of His_6_-tagged protein was carried out by use of Ni-NTA Sepharose chromatography [46]. The concentration of purified protein was quantified by BCA assays, and its purity was judged by sodium dodecyl sulfate polyacrylamide gel electrophoresis (SDS-PAGE) analysis. Similarly, *bldD* was amplified with the primers bldD-F and bldD-R (Table 2), and expressed in *E. coli* BL21 (DE3).

### Electrophoretic mobility shift assays (EMSAs)

EMSAs were carried out in the light of previously described methods [47]. Using the genomic DNA from *S. erythraea* A226 as a template, the promoter regions of *eryAI* and *ermE* were successively amplified by PCR with their respective primers (Table 2) labeled at 5′ end by the 6-isomer of carboxyfluorescein (6-FAM). Those labeled DNA fragments were independently mixed with purified His_6_-tagged SACE_7301 or BldD proteins. The binding reaction system consisted of 10 mM Tris (pH 7.5), 5 mM MgCl_2_, 50 mM EDTA, 60 mM KCl, 10 mM DTT, 10% glycerol, 150 ng labeled probes and 0.5-4 μM purified SACE_7301 or BldD. For competitive inhibition of the binding reaction, 7.5 μg unlabeled above-mentioned DNA fragments or 7.5 μg poly dIdC were added into that reaction system, respectively. After incubation on ice for 10 min, the reactants were run on an 8% TBE polyacrylamide gel (Bio-Rad) with 0.5 × TBE as a running buffer at 30 mA for 1 h.

## Additional file

Additional file 1: Figure S1. Purification of His_6_-tagged SACE _7301 and BldD. *Left lane* 116 KDa protein ladder. **Figure S2.** Time courses of dry weight of mycelia (DWM) of low-yielding *S. erythraea* strain A226, industrial overproducer WB, and their derivatives. Mean values of at least three replicates were shown, with the standard deviation indicated by error bars. **Figure S3.** Growth of A226 and A226/3×7301 treated with erythromycin. The two strains were cultured in the R5 liquid medium for 2 days by addition of four concentrations of erythromycin (40 μg/ml, 80 μg/ml, 160 μg/ml and 320 μg/ml), respectively, and their dry weights of mycelia (DWM) were successively measured. Mean values of at least three replicates were shown, with the standard deviation indicated by error bars. **Figure S4.** PCR analysis of the cassette containing 3 copies of P*ermE**-*SACE_7301* from A226/3×7301 using the primers Test-F and Test-R. The specific primers flanking above-mentioned cassette of the plasmid pSET152-3×7301 were designed as shown in Table 2. A226/3×7301 was cultured in the R5 liquid medium for 6 days without apramycin. Lanes: M: 5, 000-bp DNA ladder, +: positive control (pSET152-3×7301 as a template), -: negative control (Genomic DNA of A226 as a template), 1 to 6: Genomic DNA of A226/3×7301 cultured for 1 to 6 days, respectively, 7: Genomic DNA of the mixture of A226/7301, A226/2×7301 and A226/3×7301 with equal amount. **Figure S5.** Phylogenetic tree of SACE_7301 homologs by the neighbor-joining method. The number at each node indicates the percentage of 1000 bootstrap replications.

## Abbreviations

Er-A: Erythromycin A
*ery*: erythromycin biosynthetic gene
TFR: TetR family transcriptional regulator
PKS: Polyketide synthase
LB: Luria-Bertani medium
*tsr*: thiostrepton resistance gene
qRT-PCR: quantitative real-time PCR
IPTG: Isopropylthiogalactoside
SDS-PAGE: Sodium dodecyl sulfate polyacrylamide gel electrophoresis
EMSA: Electrophoretic mobility shift assay
6-FAM: 6-isomer of carboxyfluorescein
