# Supplementary material for: Dissecting and engineering of the TetR family regulator SACE_7301 for enhanced erythromycin production in Saccharopolyspora erythraea
Source: Microb Cell Fact. 2014 Nov 13;13:158. doi: 10.1186/s12934-014-0158-4 (PMC4258057; doi:10.1186/s12934-014-0158-4)
Supplement: Additional file 1: Figure S1. — Purification of His6-tagged SACE _7301 and BldD. Left lane 116 KDa protein ladder. Figure S2. Time courses of dry weight of mycelia (DWM) of low-yielding S. erythraea strain A226, industrial overproducer WB, and their derivatives. Mean values of at least three replicates were shown, with the standard deviation indicated by error bars. Figure S3. Growth of A226 and A226/3×7301 treated with erythromycin. The two strains were cultured in the R5 liquid medium for 2 days by addition of four concentrations of erythromycin (40 μg/ml, 80 μg/ml, 160 μg/ml and 320 μg/ml), respectively, and their dry weights of mycelia (DWM) were successively measured. Mean values of at least three replicates were shown, with the standard deviation indicated by error bars. Figure S4. PCR analysis of the cassette containing 3 copies of PermE*-SACE_7301 from A226/3×7301 using the primers Test-F and Test-R. The specific primers flanking above-mentioned cassette of the plasmid pSET152-3×7301 were designed as shown in Table 2. A226/3×7301 was cultured in the R5 liquid medium for 6 days without apramycin. Lanes: M: 5, 000-bp DNA ladder, +: positive control (pSET152-3×7301 as a template), -: negative control (Genomic DNA of A226 as a template), 1 to 6: Genomic DNA of A226/3×7301 cultured for 1 to 6 days, respectively, 7: Genomic DNA of the mixture of A226/7301, A226/2×7301 and A226/3×7301 with equal amount. Figure S5. Phylogenetic tree of SACE_7301 homologs by the neighbor-joining method. The number at each node indicates the percentage of 1000 bootstrap replications. [file 12934_2014_158_MOESM1_ESM.doc]

**Additional material files**


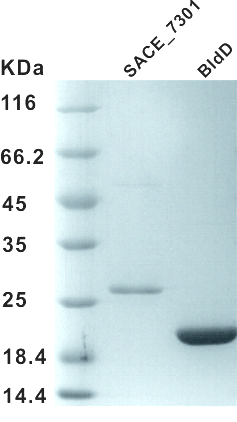


**Fig. S1 Purification of His_6_-tagged SACE _7301 and BldD. *Left lane* 116 KDa protein ladder.**


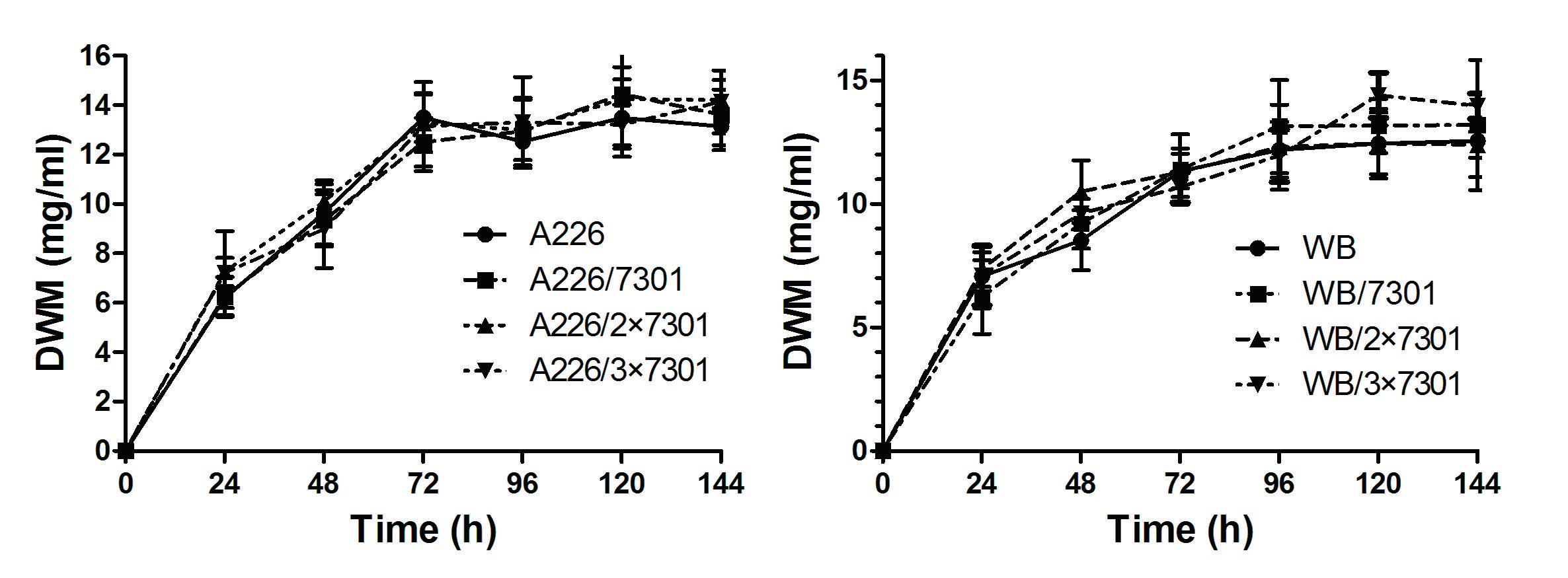


**Fig. S2 Time courses of dry weight of mycelia (DWM) of low-yielding *S. erythraea* strain A226, industrial overproducer WB, and their derivatives.** Mean values of at least three replicates were shown, with the standard deviation indicated by error bars.


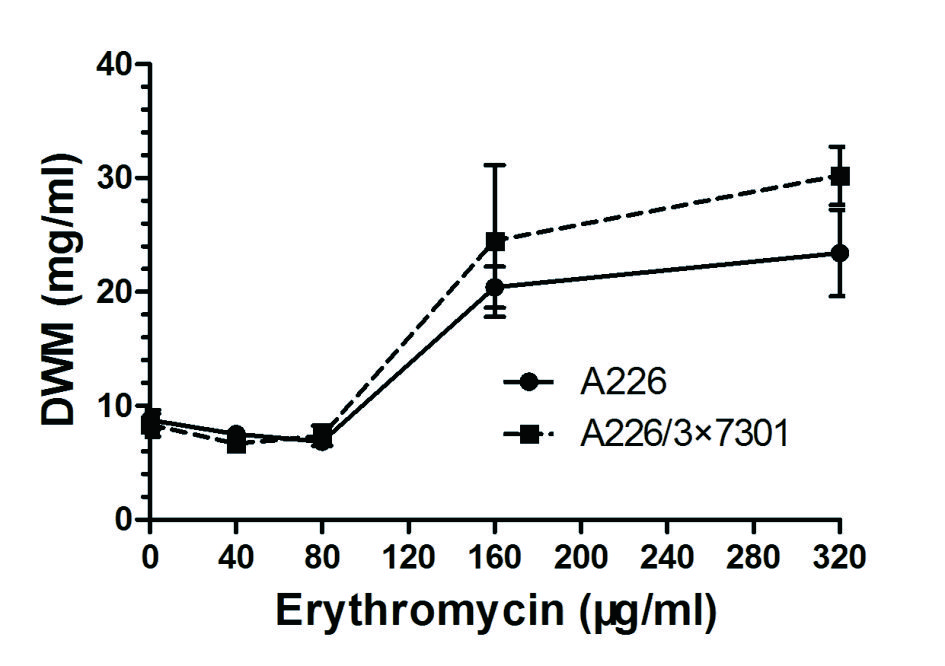


**Fig. S3 Growth of A226 and A226/3×7301 treated with erythromycin.** The two strains were cultured in the R5 liquid medium for 2 days by addition of four concentrations of erythromycin (40μg/ml, 80μg/ml, 160μg/ml and 320μg/ml), respectively, and their dry weights of mycelia (DWM) were successively measured. Mean values of at least three replicates were shown, with the standard deviation indicated by error bars.


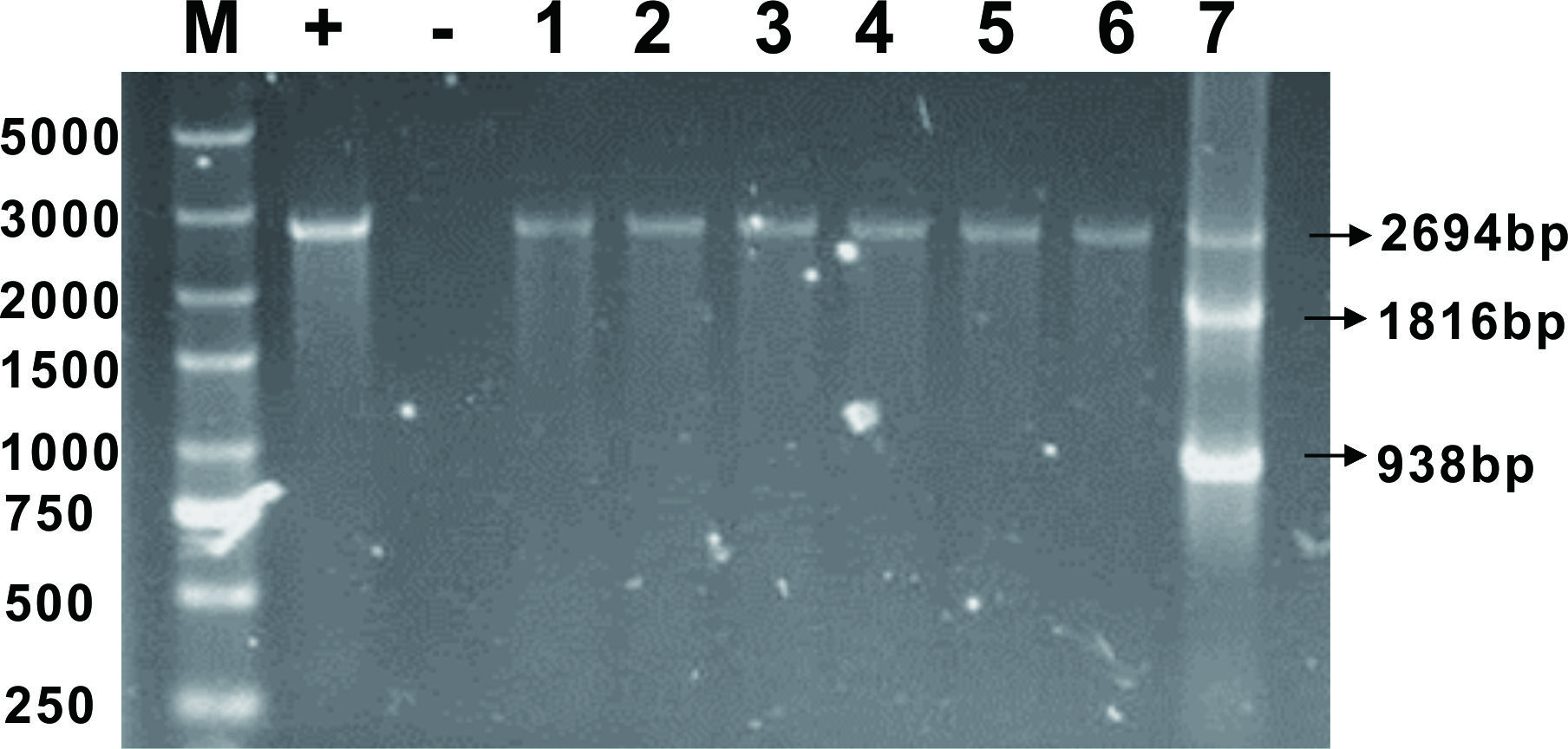


**Fig. S4 PCR analysis of the cassette containing 3 copies of P*ermE**-*SACE_7301* from A226/3×7301 using the primers Test-F and Test-R.** The specific primers flanking above-mentioned cassette of the plasmid pSET152-3×7301 were designed as shown in Table 2. A226/3×7301 was cultured in the R5 liquid medium for 6 days without apramycin . Lanes: M: 5, 000-bp DNA ladder, +: positive control (pSET152-3×7301 as a template), -: negative control (Genomic DNA of A226 as a template), 1 to 6: Genomic DNA of A226/3×7301 cultured for 1 to 6 days, respectively, 7: Genomic DNA of the mixture of A226/7301, A226/2×7301 and A226/3×7301 with equal amount .


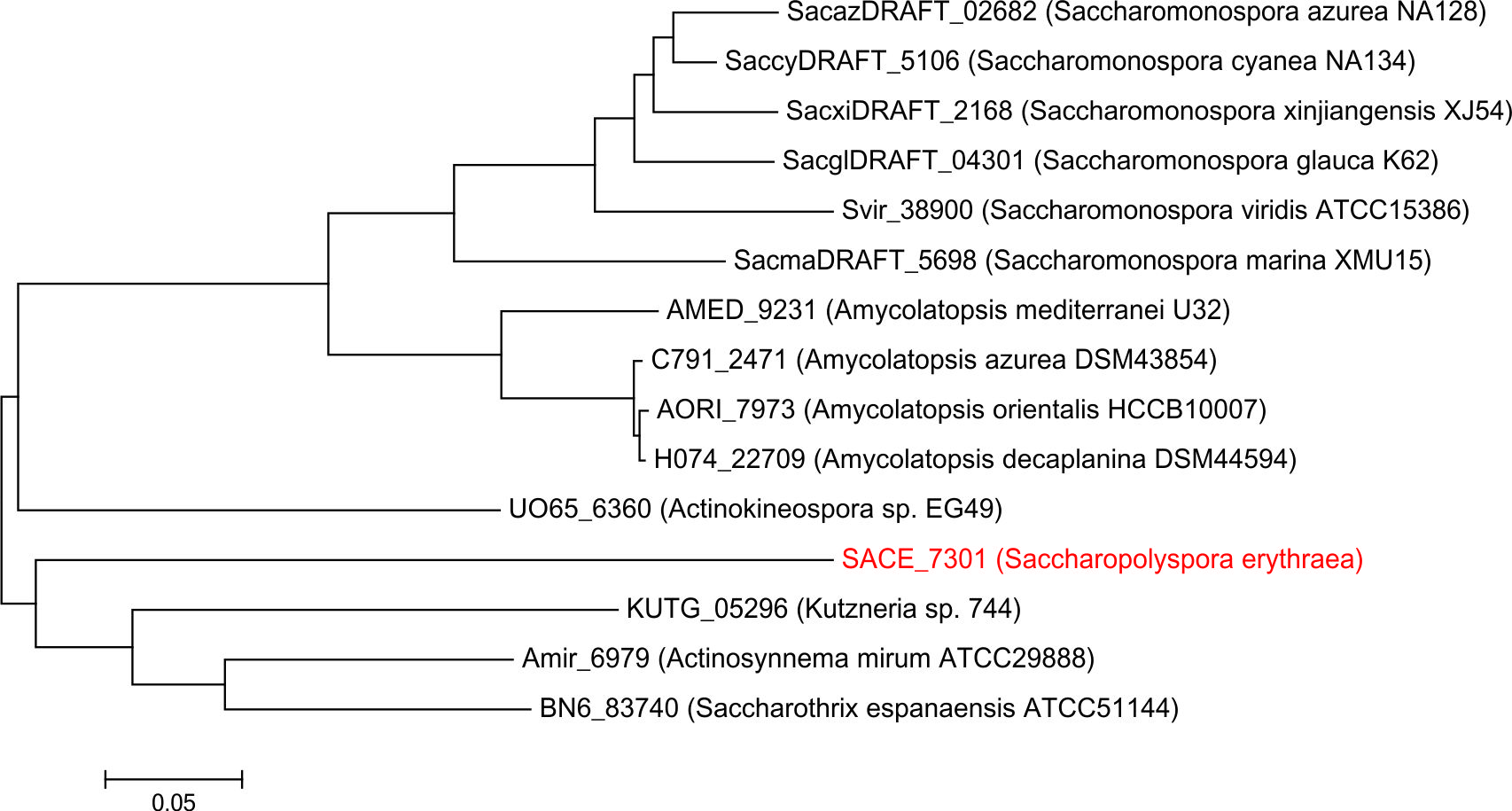


**Fig. S5 Phylogenetic tree of SACE_7301 homologs by the neighbor-joining method**. The number at each node indicates the percentage of 1000 bootstrap replications.
